# Evaluation of serum D-dimer, fibrinogen, and CA19-9 for postoperative monitoring and survival prediction in resectable pancreatic carcinoma

**DOI:** 10.1186/s12957-017-1104-9

**Published:** 2017-02-20

**Authors:** Junli Cao, Zhanzhao Fu, Liming Gao, Xin Wang, Shaohui Cheng, Xiuchao Wang, He Ren

**Affiliations:** 1 Department of Pancreatic Cancer, Key Laboratory of Cancer Prevention and Therapy, National Clinical Research Center for Cancer, Tianjin Medical University Cancer Institute and Hospital, Tianjin, 300060 People’s Republic of China; 2 Department of Oncology, First Hospital of Qinhuangdao, Hebei, 066000 People’s Republic of China; 3 Department of Intensive Medicine, Traditional Chinese Medicine Hospital of Qinhuangdao, Hebei, 066000 People’s Republic of China

**Keywords:** Pancreatic adenocarcinoma, Pancreatic cancer, Biomarkers, D-dimer, Fibrinogen, CA19-9, Postoperative monitoring, Survival prediction

## Abstract

**Background:**

We sought to investigate the efficacy of serum D-dimer, fibrinogen, and CA19-9 for postoperative monitoring and prediction of survival in patients with resectable pancreatic carcinoma (PC).

**Methods:**

One hundred and nineteen patients with resectable PC were enrolled. Serum D-dimer, fibrinogen, and CA19-9 values were analyzed before surgery and at the stages of relapse-free and progression disease.

**Results:**

D-dimer, fibrinogen, and CA19-9 were significantly higher at the active stage of PC than those at the relapse-free stage [1059.2 (1690.1) ng/ml vs 485.18 (289.84) ng/ml, (3.71 ± 0.83) g/l vs (2.75 ± 0.52) g/l, 207.2 (681.8) U/ml vs 24.5 (30) U/ml, respectively, *p <* 0.01]. Patients with elevated preoperative D-dimer had significantly shorter overall survival (18.9 ± 1.9 months vs 29.2 ± 2.6 months, *p <* 0.01) and progression-free survival (10.6 ± 1.2 months vs 20.4 ± 2.4 months, *p <* 0.01) than did those with low D-dimer. The correlation between CA19-9 values and survival depended on the threshold value of CA19-9: when the threshold value was 37 U/ml, there was no correlation between CA19-9 and survival; when the threshold value was 253.8 U/ml (median CA19-9 for the enrolled patients), patients with elevated preoperative CA19-9 had significantly shorter overall survival (19.9 ± 2. 1 months vs 29.0 ± 2. 7 months) and progression-free survival (11.5 ± 1.5 months vs 21.0 ± 2. 6 months) than did the patients with low CA19-9 (*p <* 0.01); when the threshold value was 1000 U/ml, the overall survival was 15.5 ± 2.3 months vs 28.0 ± 2.0 months and the progression-free survival 8.9 ± 1.9 months vs 19.1 ± 1.9 months (*p <* 0.01). There was no correlation between fibrinogen and overall survival (25.8 ± 2.1 months vs 21.2 ± 2.9 months; *p =* 0.096) and progression-free survival (17.8 ± 2.1 months vs 12.7 ± 1.7 months; *p =* 0.168).

**Conclusions:**

For postoperative monitoring of patients with resectable PC, D-dimer, fibrinogen, and CA19-9 may be used as markers for monitoring disease relapse, but only preoperative D-dimer could predict survival.

## Background

Pancreatic carcinoma (PC) is a leading cause of cancer-related mortality, with 5-year survival less than 8% [1, 2]. Despite substantial therapeutic progress, PC remains one of the most aggressive cancers. Radical surgery is the most effective therapy for resectable PC, but postoperative recurrence or metastatic disease are barriers to prolonged survival. For most patients, the disease progresses soon after surgery, and, unfortunately, imaging is not sufficiently sensitive to detect early recurrence or metastasis. Thus, better markers for post-resection disease monitoring are needed and are being investigated [3–7].

Abnormalities in the coagulation/fibrinolytic system, especially plasma D-dimer and fibrinogen, have been identified in cancer patients. D-dimer is a degradation product of crosslinked fibrin and a sign of fibrinolytic hyperfunction in vivo. D-dimer has been reported to be a marker of various cancers [8–10]. Fibrin and fibrinogen are identified as major components of the tumor stroma enveloping tumor cells. Plasma D-dimer values and fibrinogen have been reported to be prognostic factors for PC [11–13], but most of these reports focused on metastatic PC, and the results were conflicting [12, 14]. CA 19-9, or sialyl Lewis-a (sLea), is a glycolipid and an O-linked glycoprotein expressed on the surface of cancer cells. CA19-9 is derived from an aberrant pathway during production of its normal counterpart, disialyl Lewis-a. CA19-9 is widely used as biomarker for PC and can be combined with other markers, despite having several limitations [3–7]. So, we investigated whether serum D-dimer, fibrinogen, and CA19-9 are reliable markers for postoperative monitoring and prediction of patient survival for resectable PC.

## Methods

### Patients

Subjects (*N* = 119) were included who met the following criteria: (1) histological confirmation of the diagnosis; (2) tumor resectability, confirmed intraoperatively and by histopathological examinations; (3) radical surgery performed; (4) plasma D-dimer, fibrinogen, and CA19-9 values were measured before surgery (samples taken within 7 days before surgery) and regularly after surgery (most patients were followed every 1 month in the first year and then every 3 months but some had shorter or longer follow-ups); (5) The subjects also had adequate clinical information for survival analysis in patient records and death that was tumor-related (Table 1). Patients with apparent inflammatory diseases, a history of thrombosis or treatment with drugs that might influence the coagulation/fibrinolytic system, or severe jaundice that might influence CA19-9 were excluded. Also, 30 samples from healthy individuals and 40 samples from patients with serous pancreatic cystadenoma were collected as controls. The “relapse-free stage” was defined as no new lesions found by CT or MRI. “Progressive disease” was defined as new lesions found by CT or MRI or biopsy.

**Table 1:** Relationships between clinicopathological characteristics and preoperative plasma D-dimer, fibrinogen, and CA19-9 values in 119 PC patients

Variable	Total (*n*)	D-dimer (ng/ml)			Fibrinogen (g/l)			CA19-9 (U/ml)		
		≤500^a^	>500	*p* value	≤4^a^	>4	*p* value	≤37^a^	>37	*p* value
Sex	119			0.809			0.709			0.323
Male	70	37	33		48	22		15	55	
Female	49	27	22		32	17		7	42	
Age (years)	119			0.580			0.352			0.071
≤60	66	34	32		42	24		16	50	
>60	53	30	23		38	15		6	47	
Tumor size	119			0.514			0.926			0.718
≤4 cm	94	52	42		63	31		18	76	
>4 cm	25	12	13		17	8		4	21	
TNM	119			0.116			0.512			0.564
I	32	21	11		23	9		7	25	
II	87	43	44		57	30		15	72	
Lymphatic metastasis	119			0.678			0.674			0.036
Negative	95	52	43		63	32		14	81	
Positive	24	12	12		17	7		8	16	
Tumor differentiation	119			0.266			0.256			0.842
1–2 grade	90	51	39		63	27		17	73	
3 grade	29	13	16		17	12		5	24	

^a^Normal upper limit

### Biomarker measurements

Plasma D-dimer was measured with Innovance D-dimer immunoassays with the Vidas D-dimer kit (VIDAS DD, France); fibrinogen was measured by immunoturbidimetry. CA19-9 was measured with a chemiluminescent immunoassay. All blood samples were collected in the early morning, fasting. Normal reference values for plasma D-dimer, fibrinogen, and CA19-9 were 0–500 ng/ml, 2–4.0 g/l, and 0–37 U/ml, respectively.

### Statistical analysis

Measurements were reported as means ± SEM or as medians (interquartile range), depending on the distribution of data. An independent sample Student’s *t* test and a nonparametric test were used to make comparisons between groups. A Chi-squared test was used to evaluate the association between clinicopathological measurements and D-dimer, fibrinogen, or CA19-9 values. Survival outcomes were analyzed with a Kaplan-Meier method, and comparisons between groups were made using a log-rank test. To establish a COX regression model analysis of independent factors for survival of patients with PC, statistical analyses were performed with SPSS17.0 software (*p* values ≤0.05 were considered statistically significant).

## Results

### Preoperative plasma D-dimer, fibrinogen, and CA19-9 values in PC patients

The median (interquartile range) of preoperative plasma D-dimer and CA19-9 were 450.0 (688.3) ng/ml and 253.8 (1188) U/ml, respectively. Fibrinogen was (3.59 ± 0.84) g/l. Plasma D-dimer values were above 500 ng/ml in 55 (46.2%) patients; 32.8% of patients had hyperfibrinogemia; and 100 (84.0%) had higher than normal CA19-9. In healthy controls, the median (interquartile range) of plasma D-dimer and CA19-9 were 109.89 (124.09) ng/ml and 12.27(10.14) U/ml, respectively, and fibrinogen was (2.49 ± 0.37) g/l. For patients with serous pancreatic cystadenoma, the median (interquartile range) of plasma D-dimer and CA19-9 were 218.86 (227.27) ng/ml and 28.73(28.9) U/ml, respectively, and fibrinogen was (2.78 ± 0.50) g/l. Three biomarkers were higher in patients with serous pancreatic cystadenoma than healthy controls, but all were within the normal range. A Chi-squared test showed that CA19-9 was a more sensitive marker for PC than D-dimer or fibrinogen (*χ* ^2^ = 37.46 and 64.36, respectively, *p* < 0.01). There were no significant correlations between clinicopathological characteristics (sex, age, tumor size, TNM stage, lymphatic metastasis, tumor differentiation) and plasma D-dimer or fibrinogen values (*p >* 0.05). However, among the subset of patients who had lymphatic metastases, CA19-9 values were significantly higher than in those who did not have metastases (*p =* 0.036).

### Postoperative plasma D-dimer, fibrinogen, and CA19-9 values as biomarkers for postoperative monitoring of PC

As shown in Table 2, plasma D-dimer, fibrinogen, and CA19-9 increased significantly when disease was progressive (*p* < 0.001). Sensitivities of D-dimer, fibrinogen, and CA19-9 were 82.6, 40.4, and 79.8%, respectively.

**Table 2:** Plasma D-dimer, fibrinogen, and CA19-9 at various stages of PC

Serum test	Relapse-free stage	Progressive disease	z/t	*p*
D-dimer (ng/ml)	485.18 (289.84)	1059.2 (1690.1)	−7.490	<0.001
Fibrinogen (g/l)	2.75 ± 0.52	3.71 ± 0.83	−10.228	<0.001
CA19-9 (U/ml)	24.5 (30)	207.2 (681.8)	−8.669	<0.001

Eight patients were excluded because they also had deep vein thrombosis, and two were excluded for serious infections. Samples from patients considered at relapse-free stage were extracted 1.8 months (median) before progressive. Samples from patients considered progressive disease were extracted 5 days (median) before progressive disease

### Preoperative plasma D-dimer as a biomarker for survival prediction

Patients were followed from 3 to 66 months postoperatively. Overall survival (OS) was defined as the interval between the date of surgery and either the time of death due to PC or the last follow-up. Progression free survival (PFS) was defined as the interval between the date of surgery and either the time of disease progression or the last follow-up. Disease progression was confirmed by imaging examination or pathological examination.

**Fig. 1:**
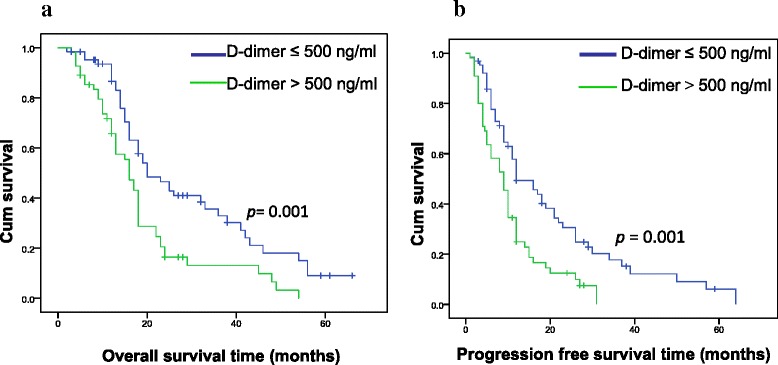
Kaplan-Meier curves for OS (**a**) and for PFS (**b**) for PC patients stratified by preoperative plasma D-dimer

**Fig. 2:**
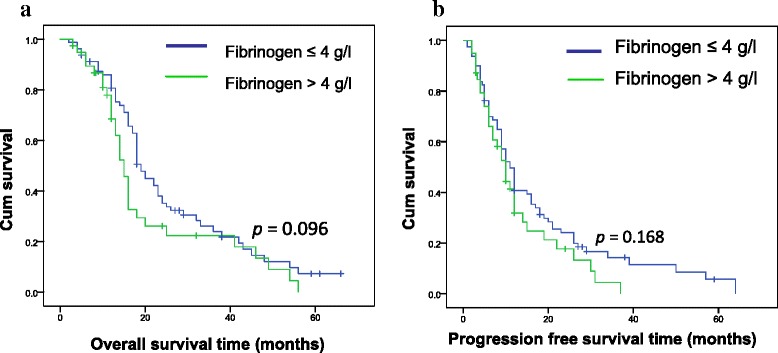
Kaplan-Meier curves for OS (**a**) and for PFS (**b**) for PC patients stratified by preoperative plasma fibrinogen

Figure 1 illustrates that patients with elevated preoperative D-dimer values (>500 ng/ml) had significantly shorter OS (18.9 ± 1.9 months vs 29.2 ± 2. 6 months; *p* = 0.001) and PFS (10.6 ± 1. 2 months vs 20.4 ± 2.4 months; *p* = 0.001) than did those with lower (≤500 ng/ml) D-dimer. In contrast, as illustrated in Fig. 2, there was no correlation between preoperative fibrinogen and OS (25.8 ± 2.1 months vs 21.2 ± 2.9 months, *p =* 0.096), and PFS (17.8 ± 2.1 months vs 12.7 ± 1.7 months, *p =* 0.168).

D-dimer was abnormally high in 48 patients, and CA19-9 was elevated in 38 patients who were considered at relapse-free stage according to imaging. Statistical analysis showed that abnormally higher D-dimer was associated with shorter OS (19.2 ± 2.5 months vs 27.2 ± 2.4 months, *p* = 0.017), but there was no difference between abnormal and normal CA19-9 (21.5 ± 2.9 months vs 25.1 ± 2.3 months, *p* = 0.265). Elevated D-dimer and CA19-9 values were associated with shorter PFS (13.2 ± 2.3 months vs 17.7 ± 2.3 months and 12.4 ± 2.3 months vs 17.8 ± 2.3 months, *p* = 0.065 and 0.074, respectively), but these differences were not significant.

**Fig. 3:**
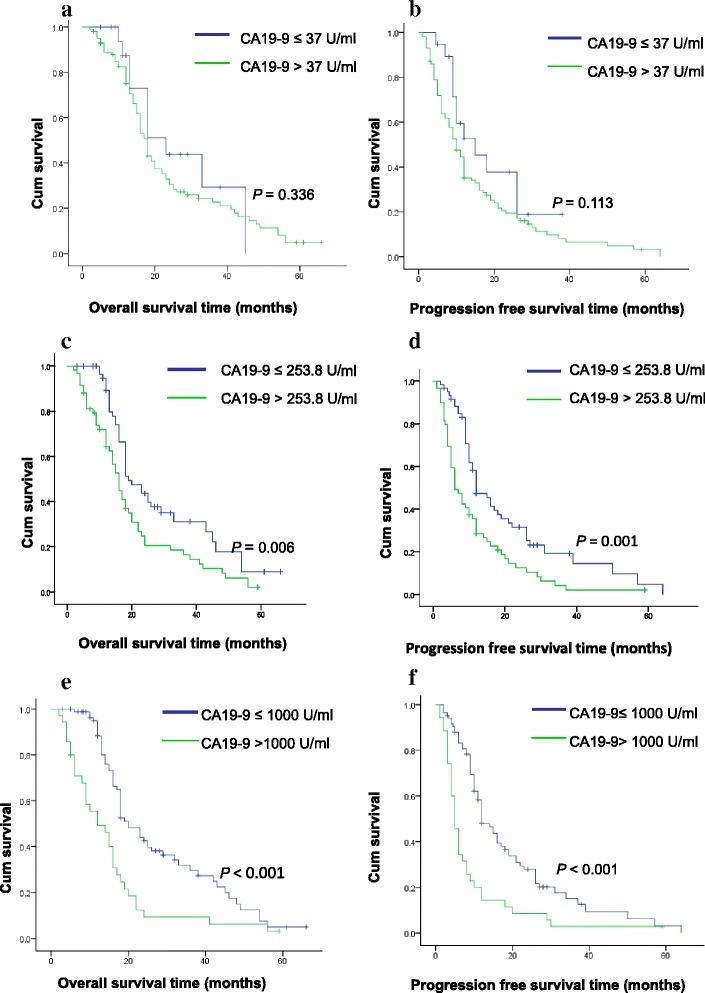
Kaplan-Meier curves for OS and PFS for PC patients stratified by preoperative CA19-9. **a**, **b** Threshold value of 37 U/ml. **c**, **d** Threshold value of 253.8 U/ml. **e**, **f** Threshold value of 1000 U/ml

Table 3 and Fig. 3 illustrate relationships between preoperative CA19-9 values and survival, and OS and PFS depended on the CA19-9 threshold value. When the threshold value was 37 U/ml, it did not affect the survival. If the threshold value was 253.8 or 1000 U/ml, patient survival was negatively correlated with CA19-9 values. Tables 4 and 5 show that preoperative D-dimer was an independent risk factor for PFS for patients and that preoperative D-dimer and CA19-9 were independent factors affecting OS.

**Table 3:** Comparisons of survival and preoperative CA19-9 in PC patients

Variable	CA19-9 (U/ml)					
	≤37	>37	≤253.8	>253.8	≤1000	>1000
OS (m)	27.8 ± 3.9	23.8 ± 1.8	29.0 ± 2.7	19.9 ± 2.1	28.0 ± 2.0	15.5 ± 2.3
PFS (m)	18.8 ± 2.9	15.1 ± 1.6	21.0 ± 2.6	11.5 ± 1.5	19.1 ± 1.9	8.9 ± 1.9

**Table 4:** COX regression model analysis of PFS for PC patients

	B	SE	Wald	Sig.	Exp (B)
Preoperative D-dimer	0.000	0.000	12.780	0.000	1.000
Preoperative fibrinogen	0.097	0.145	0.443	0.506	1.102
Preoperative CA19-9	0.000	0.000	2.189	0.139	1.000

**Table 5:** COX regression model analysis of OS for PC patients

	B	SE	Wald	Sig.	Exp (B)
Preoperative D-dimer	0.000	0.000	6.250	0.012	1.000
Preoperative fibrinogen	0.185	0.162	1.298	0.255	1.203
Preoperative CA19-9	0.000	0.000	5.071	0.024	1.000

## Discussion

We investigated whether serum D-dimer, fibrinogen, and CA19-9 are reliable biomarkers for postoperative monitoring and prediction of survival in patients with resectable PC. We found that plasma D-dimer values could predict OS and PFS of PC and CA19-9 correlated with OS and PFS but only when the threshold value exceeded normal values and there was no correlation between fibrinogen and OS or PFS. Also, D-dimer, fibrinogen, and CA19-9 were significantly higher with active disease than when the cancer is at a relapse-free stage. D-dimer and CA19-9 were abnormally high in some patients who were considered to be at a relapse-free stage according to CT or MRI scan. Furthermore, patients with abnormally high D-dimer had shorter OS than those with normal D-dimer. Likely, rising D-dimer and CA19-9 values may predict early postoperative tumor recurrence or progression of PC, but more work is required to confirm this.

Some studies have indicated that the coagulation/fibrinolytic system is activated in cancer patients and it may contribute to cancer progression [15, 16]. Thus, tumor-related degradation products of the coagulation and fibrinolytic system have been proposed to predict tumor load and prognosis [15, 16]. Plasma D-dimer is a procoagulation factor that may reflect the presence of micrometastases or circulating tumor cells, which may be responsible for tumor recurrence [17]. Hyperfibrinogenemia can enhance the sustained adherence of tumor cell emboli in the vasculature of target organs, helping to establish metastases [18]. Because of a likely involvement of the coagulation/fibrinolytic system in cancer, we studied whether D-dimer and fibrinogen are useful biomarkers for postoperative monitoring and survival prediction for resectable PC. We found that although plasma D-dimer and fibrinogen were higher when disease was active, only D-dimer values were correlated with OS and PFS.

Our observations on the coagulation/fibrinolytic system in PC can be compared with other reports, although this is difficult because of differences in clinical circumstances and study objectives. Stender’s group [19] reported that D-dimer is prognostic for non-resectability of PC, and Durcynski et al. [20, 21] reported that high D-dimer values may predict unresectability even when imaging studies predicted resectability. Liu et al. [12] indicated that plasma D-dimer and platelet counts and treatment response are independent prognostic factors for OS which was consistent with our results. Tas’ group [14] reported that serum D-dimer was elevated in patients with metastatic pancreatic adenocarcinoma. Sun et al. [22] found that pretreatment plasma D-dimer values were potentially prognostic in pancreatic adenocarcinoma even in the absence of venous thromboembolism. Most current study objectives are focused on unresectable or metastatic pancreatic adenocarcinoma which differ from our objectives. Thus, D-dimer measurements may have clinical utility for managing PC.

Studies of fibrinogen in PC, such as those by Qi and colleagues [13] suggest that hyperfibrinogen with associated systemic inflammatory response was predictive of poor prognosis in advanced PC. Wang’s group [23] reported that pretreatment platelet and fibrinogen correlated with tumor progression, metastases, and OS for patients with PC. Guo’s group [11] reported that elevated fibrinogen may predict metastases. Our results appear to contradict these, which may be explained by patients at different disease stages. Thus, the status of fibrinogen with respect to clinical assessment of PC is uncertain.

Serum CA19-9 has been used as a marker for diagnosis and therapeutic monitoring of PC, but few studies have investigated the value of CA19-9 for predicting survival in resectable PC. We found that serum CA19-9 could not predict survival in PC when the threshold value was set at 37 U/ml, which conflicts with the conclusion from Ballehaninna’s group [24], but this warrants study because we included 19 patients with CA19-9 ≤ 37 U/ml. When we analyzed survival using CA19-9 values of >1000 or 253.8 U/ml (median CA19-9 of all subjects), the patients had significantly shorter OS and PFS. Thus, it appears that if an appropriate threshold value of CA19-9 is used, CA19-9 can be a predictor of survival in resectable PC. This opinion is corroborated by work by Qi’s group [13], who reported that CA19-9 ≥ 1000 U/ml were prognostic for poor OS. Matsumoto et al. [25] reported that preoperative CA19-9 ≥ 300 U/ml values are independent risk factors for early recurrence of PC. In other work in PC, early decreases in CA19-9 reportedly predicted favorable outcomes in advanced disease [26]. In this study, we found that positive expression of CA19-9 was greater than that of D-dimer and fibrinogen. This finding may also reflect the fact that activation of the coagulation/fibrinolytic system mostly occurs in advanced disease, and most of our patients had early-stage disease. In addition, most of our patients had small tumors and a low rate of lymph node metastases.

Our study is limited as are other studies to identify cancer biomarkers. Scara’s group [27] reported that although many putative biomarkers for PC have been proposed, most lack validation and none has been shown to possess the requisite sensitivity and specificity to be introduced into clinical use.

## Conclusions

For postoperative monitoring of patients with resectable PC, fibrinogen and CA19-9 may be used as markers, but only preoperative D-dimer predicts survival. This information may be useful for the care and counseling of patients who undergo surgery for pancreatic adenocarcinoma.

## Abbreviations

OS: Overall survival
PC: Pancreatic carcinoma
PFS: Progression free survival
